# Pallidal versus subthalamic deep-brain stimulation for meige syndrome: a retrospective study

**DOI:** 10.1038/s41598-021-88384-4

**Published:** 2021-04-22

**Authors:** Jiayu Liu, Hu Ding, Ke Xu, Ruen Liu, Dongliang Wang, Jia Ouyang, Zhi Liu, Zeyu Miao

**Affiliations:** Department of Neurosurgery, Peking University People’s Hospital, 11th Xizhimen South St., Beijing, 100044 China

**Keywords:** Neurology, Neurological disorders

## Abstract

Deep-brain stimulation (DBS) is an effective treatment for patients with Meige syndrome. The globus pallidus interna (GPi) and the subthalamic nucleus (STN) are accepted targets for this treatment. We compared 12-month outcomes for patients who had undergone bilateral stimulation of the GPi or STN. Forty-two Asian patients with primary Meige syndrome who underwent GPi or STN neurostimulation were recruited between September 2017 and September 2019 at the Department of Neurosurgery, Peking University People’s Hospital. The primary outcome was the change in motor function, including the Burke–Fahn–Marsden Dystonia Rating Scale movement (BFMDRS-M) and disability subscale (BFMDRS-D) at 3 days before DBS (baseline) surgery and 1, 3, 6, and 12 months after surgery. Secondary outcomes included health-related quality of life, sleep quality status, depression severity, and anxiety severity at 3 days before and 12 months after DBS surgery. Adverse events during the 12 months were also recorded. Changes in BFMDRS-M and BFMDRS-D scores at 1, 3, 6, and 12 months with DBS and without medication did not significantly differ based on the stimulation target. There were also no significant differences in the changes in health-related quality of life (36-Item Short-Form General Health Survey) and sleep quality status (Pittsburgh Sleep Quality Index) at 12 months. However, there were larger improvements in the STN than the GPi group in mean score changes on the 17-item Hamilton depression rating scale (− 3.38 vs. − 0.33 points; *P* = 0.014) and 14-item Hamilton anxiety rating scale (− 3.43 vs. − 0.19 points; *P* < 0.001). There were no significant between-group differences in the frequency or type of serious adverse events. Patients with Meige syndrome had similar improvements in motor function, quality of life and sleep after either pallidal or subthalamic stimulation. Depression and anxiety factors may reasonably be included during the selection of DBS targets for Meige syndrome.

## Introduction

Meige syndrome is a rare adult-onset dystonia characterized by blepharospasm, oromandibular dystonia and cervical dystonia^1^. Deep-brain stimulation (DBS) is used to treat dystonia by implanting stimulating electrodes in specific parts of the brain and by continuously emitting electrical signals to suppress abnormal electrical discharges and regulate motor circuits^2^. Although the globus pallidus interna (GPi) is the most commonly used target of DBS in the treatment of Meige syndrome^3,4^, our recent study has demonstrated the efficacy and safety of the subthalamic nucleus (STN) as a target of DBS^5^. This study was designed to compare the outcomes of bilateral neurostimulation of pallidal stimulation (GPi-DBS) with that of subthalamic stimulation (STN-DBS). To our knowledge, this is the largest study assessing and comparing the efficacy of stimulating these targets in patients with Meige syndrome.

## Results

### Patients

A total of 42 patients with Meige syndrome underwent either bilateral pallidal stimulation (21 patients) or bilateral subthalamic stimulation (21 patients). The clinical characteristics of the two groups of patients were similar at baseline in terms of age (58.67 ± 6.76 years in the GPi group vs. 59.57 ± 9.42 years in the STN group); female sex (66.7% vs. 81.0%); medication for Meige syndrome (42.9% vs. 28.6%); botulinum toxin treatment (42.9% vs. 23.8%); family history of dystonia (9.5% vs. 9.5%) and disease duration (6.21 ± 6.06 years vs. 6.39 ± 6.09 years). Baseline functional status (BFMDRS-M and BFMDRS-D scores) and quality of life (SF-36, HAMD, HAMA and PSQI scores) characteristics did not significantly differ between the two groups (Table 1).

**Table 1:** Characteristics of the Patients at Baseline.

Characteristic*	Pallidal stimulation	Subthalamic stimulation	*P* value^†^
**Demographic or clinical**			
Age-yr	58.67 ± 6.76	59.57 ± 9.42	0.722
Female sex—no. (%)	14 (66.7)	17 (81.0)	0.292
Medication for Meige Syndrome—no. (%)	9 (42.9)	6 (28.6)	0.334
Botulinum toxin treatment—no. (%)	9 (42.9)	5 (23.8)	0.190
Family history of dystonia—no. (%)	2 (9.5)	2 (9.5)	0.697
Disease duration—yr	6.21 ± 6.06	6.39 ± 6.09	0.925
**Functional status**			
BFMDRS-M (range, 0–40)^§^	11.19 ± 5.27	9.64 ± 4.11	0.295
Eye (range, 0–8)	5.24 ± 2.77	5.79 ± 2.06	0.472
Mouth (range, 0–8)	3.95 ± 2.56	2.90 ± 2.42	0.181
Speech & swallowing (range, 0–16)	1.38 ± 2.09	0.57 ± 1.91	0.197
Neck (range, 0–8)	0.62 ± 1.63	0.38 ± 1.36	0.610
BFMDRS-D (range, 0–30)^§^	4.19 ± 3.56	4.14 ± 2.50	0.960
Speech (range, 0–4)	0.57 ± 0.68	0.29 ± 0.46	0.118
Writing (range, 0–4)	0.90 ± 1.00	1.19 ± 0.98	0.354
Feeding (range, 0–4)	0.57 ± 0.60	0.81 ± 051	0.173
Eating and swallowing (range, 0–4)	0.43 ± 0.75	0.05 ± 0.22	0.147
Hygiene (range, 0–4)	0.24 ± 0.44	0.29 ± 0.46	0.733
Dressing (range, 0–6)	0.14 ± 0.36	0.05 ± 0.22	0.305
Walking (range, 0–4)	1.33 ± 1.39	1.48 ± 0.93	0.697
**Quality of life**			
SF-36 scores ^¶^			
General health (range, 0–100)	25.48 ± 12.14	27.14 ± 10.67	0.639
Physical function (range, 0–100)	77.38 ± 21.25	72.62 ± 22.00	0.480
Role—physical (range, 0–100)	38.10 ± 42.29	40.48 ± 48.40	0.866
Role—emotional(range, 0–100)	63.49 ± 40.70	61.90 ± 39.84	0.899
Social function (range, 0–100)	51.79 ± 20.27	45.83 ± 14.97	0.285
Body pain (range, 0–100)	100.00 ± 0.00	97.85 ± 9.82	0.323
Vitality (range, 0–100)	39.05 ± 14.80	38.10 ± 7.98	0.797
Mental health(range, 0–100)	41.52 ± 17.69	46.29 ± 10.40	0.294
17-item Hamilton Depression Rating Scale (range, 0–52)^§^	12.05 ± 6.25	9.29 ± 4.01	0.096
14-item Hamilton anxiety rating scale (range, 0–56)^§^	14.76 ± 6.69	11.14 ± 5.20	0.060
Pittsburgh Sleep Quality Index (range, 0–21)^§^	13.48 ± 4.85	13.05 ± 4.49	0.521

*Plus–minus values are means ± SD.

^†^* P* value s were calculated with the use of Student’s t-test or analysis of variance for continuous variables and Fisher’s exact test for categorical variables.

^§^A higher score indicates worse functioning.

^¶^A higher score indicates better functioning.

### Motor function

The primary outcome, the change in BFMDRS-M scores at 1, 3, 6, and 12 months with DBS and without medication, did not significantly differ based on the stimulation target.

**Figure 1:**
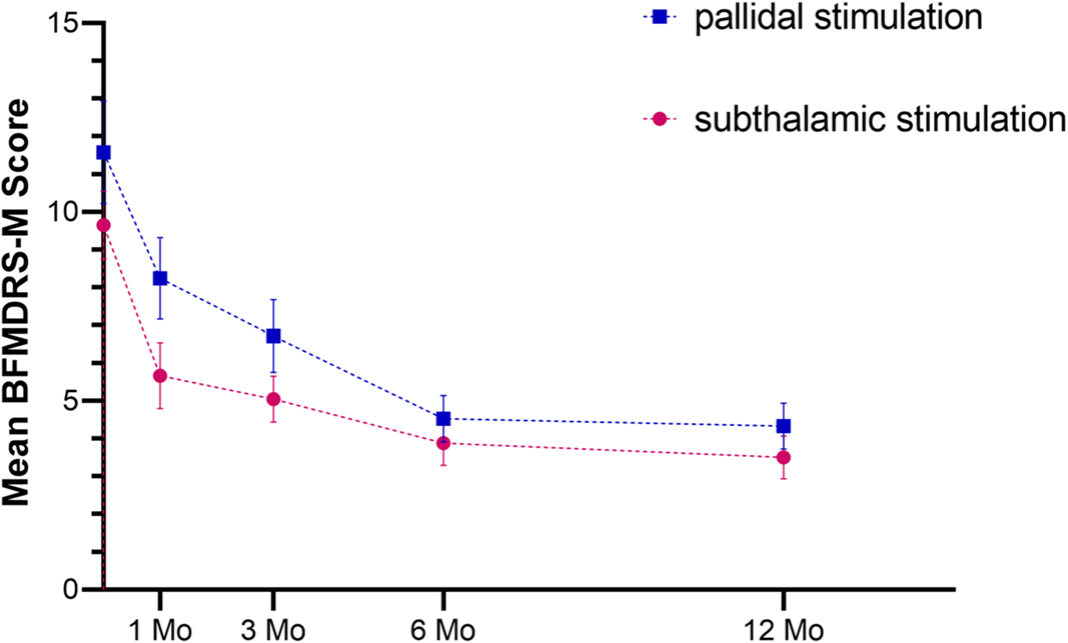
Burke–Fahn–Marsden Dystonia Rating Scale movement subscale scores (BFMDRS-M; scores range from 0 to 40, with higher scores indicating greater impairment) at baseline and throughout the 12-month study period. The changes in the BFMDRS-M scores at 1, 3, 6, and 12 months with deep-brain stimulation and without medication did not significantly differ based on the stimulation target. I bars indicate the standard error of the mean.

At 1 month after surgery, the mean BFMDRS-M scores (highest possible score, 40) improved by 26.4% in the GPi group (from 11.19 ± 5.27 to 8.24 ± 4.95) and by 41.2% in the STN group (from 9.64 ± 4.11 to 5.67 ± 3.98). At 3 months, scores improved by 40.0% in the GPi group (from 11.19 ± 5.27 to 6.71 ± 4.40) and by 47.6% in the STN group (from 9.64 ± 4.11 to 5.05 ± 2.78) (Fig. 1, Table 2). At 6 months, scores improved by 59.6% in the GPi group (from 11.19 ± 5.27 to 4.52 ± 2.83) and by 59.8% in the STN group (from 9.64 ± 4.11 to 3.88 ± 2.69). At 12 months, scores improved by 61.3% in the GPi group (from 11.19 ± 5.27 to 4.33 ± 2.78) and by 63.7% in the STN group (from 9.64 ± 4.11 to 3.50 ± 2.60) (Fig. 1, Table 3). During the 12 months of follow-up, there were also no significant differences in improvements on the four subscale scores of the BFMDRS-M between the two groups. At 12 months after surgery, the mean score on the eye subscale (highest possible score, 8) improved by 65.1% in the GPi group (from 5.24 ± 2.77 to 1.83 ± 1.30) and by 64.2% in the STN group (from 5.79 ± 2.06 to 2.07 ± 1.44); the mean score on the mouth subscale (highest possible score, 8) improved by 59.0% in the GPi group (from 3.95 ± 2.56 to 1.62 ± 1.21) and by 59.0% in the STN group (from 2.90 ± 2.42 to 1.19 ± 1.49); the mean score on the speech & swallowing subscale (highest possible score, 16) improved by 62.3% in the GPi group (from 1.38 ± 2.09 to 0.52 ± 0.98) and by 91.2% in the STN group (from 0.57 ± 1.91 to 0.05 ± 0.22); and the mean score on the neck subscale (highest possible score, 8) improved by 41.9% in the GPi group (from 0.62 ± 1.63 to 0.36 ± 0.94) and by 50.0% in the STN group (from 0.38 ± 1.36 to 0.19 ± 0.60).

**Table 2:** Primary Outcome at 1 and 3 Months.

Characteristic*	1 months^‡^		3 months^‡^		Mean change at 1 months from baseline (95% CI)			Mean change at 3 months from baseline (95% CI)		
	Pallidal stimulation	Subthalamic stimulation	Pallidal stimulation	Subthalamic stimulation	Pallidal stimulation	Subthalamic stimulation	*P* value	Pallidal stimulation	Subthalamic stimulation	*P* value
BFMDRS-M (range, 0–40)^§^	8.24 ± 4.95	5.67 ± 3.98	6.71 ± 4.40	5.05 ± 2.78	− 3.33 (− 4.78 to − 1.79)	− 3.98 (− 5.51 to − 2.44)	0.572	− 4.86 (− 6.40 to − 3.31)	− 4.60 (− 6.13 to − 3.06)	0.828
Eye(range, 0–8)	3.40 ± 2.31	3.36 ± 1.94	2.64 ± 1.90	2.86 ± 1.44	− 1.83 (− 2.71 to − 0.95)	− 2.43 (− 3.34 to − 1.52)	0.337	− 2.60 (− 3.50 to − 1.69)	− 2.93 (− 3.80 to − 2.06)	0.574
Mouth(range, 0–8)	3.07 ± 2.29	1.88 ± 2.00	2.40 ± 1.97	1.81 ± 1.68	− 0.88 (− 1.52 to − 0.24)	− 1.02 (− 1.73 to − 0.32)	0.774	− 1.55 (− 2.26 to − 0.84)	− 1.10 (− 1.85 to − 0.34)	0.424
Speech & Swallowing(range, 0–16)	1.29 ± 1.79	0.24 ± 0.89	1.19 ± 1.75	0.19 ± 0.87	− 0.10 (− 0.29 to 0.10)	− 0.33 (− 1.03 to 0.36)	0.506	− 0.19 (− 0.46 to 0.08)	− 0.38 (− 1.18 to 0.41)	0.649
Neck(range, 0–8)	0.29 ± 0.77	0.19 ± 0.87	0.29 ± 0.77	0.19 ± 0.60	− 0.33 (− 0.83 to 0.17)	− 0.19 (− 0.46 to 0.08)	0.621	− 0.33 (− 0.83 to 0.17)	− 0.19 (− 0.59 to 0.21)	0.657
BFMDRS-D (range, 0–30)^§^	2.24 ± 2.02	1.81 ± 1.86	1.67 ± 1.62	1.38 ± 1.60	− 2.14 (− 3.35 to − 0.94)	− 2.33 (− 3.34 to − 1.32)	0.810	− 2.71 (− 3.98 to − 1.45)	− 2.76 (− 3.83 to − 1.70)	0.956
Speech (range, 0–4)	0.43 ± 0.60	0.10 ± 0.30	0.38 ± 0.59	0.14 ± 0.36	− 0.14 (− 0.31 to 0.02)	− 0.19 (− 0.37 to − 0.01)	0.715	− 0.19 (− 0.37 to − 0.01)	− 0.14 (− 0.36 to 0.07)	0.748
Writing (range, 0–4)	0.48 ± 0.60	0.48 ± 0.60	0.33 ± 0.58	0.29 ± 0.46	− 0.48 (− 0.82 to − 0.13)	− 0.71 (− 1.13 to − 0.30)	0.329	− 0.62 (− 0.99 to − 0.25)	− 0.90 (− 1.33 to − 0.48)	0.329
Feeding (range, 0–4)	0.29 ± 0.46	0.43 ± 0.51	0.19 ± 0.40	0.33 ± 0.48	− 0.33 (− 0.55 to—0.11)	0.38 (− 0.65 to − 0.11)	0.771	− 0.43 (− 0.66 to − 0.20)	− 0.48 (− 0.79 to − 0.17)	0.771
Eating and swallowing (range, 0–4)	0.38 ± 0.74	0.05 ± 0.22	0.29 ± 0.64	0.00 ± 0.00	− 0.05 (− 0.15 to 0.05)	0.00 (− 0.14 to 0.14)	0.576	− 0.14 (− 0.36 to 0.07)	− 0.05 (− 0.15 to 0.05)	0.428
Hygiene (range, 0–4)	0.00 ± 0.00	0.05 ± 0.22	0.00 ± 0.00	0.05 ± 0.22	− 0.24 (− 0.44 to − 0.04)	− 0.24 (− 0.44 to − 0.04)	1.000	− 0.24 (− 0.44 to − 0.04)	− 0.24 (− 0.44 to − 0.04)	1.000
Dressing (range, 0–6)	0.00 ± 0.00	0.00 ± 0.00	0.00 ± 0.00	0.00 ± 0.00	− 0.14 (− 0.31 to 0.02)	− 0.05 (− 0.15 to 0.05)	0.329	− 0.14 (− 0.31 to 0.02)	− 0.05 (− 0.15 to 0.05)	0.329
Walking (range, 0–4)	0.67 ± 0.73	0.71 ± 0.78	0.48 ± 0.60	0.57 ± 0.68	− 0.76 (− 1.26 to 0.27)	− 0.76 (− 1.11 to − 0.41)	1.000	− 0.95 (− 1.48 to − 0.42)	− 0.90 (− 1.22 to − 0.59)	0.880

*Plus–minus values are means ± SD.

^‡^Assessments with DBS on.

^§^A higher score indicates worse functioning.

**Table 3:** Primary Outcome at 6 and 12 Months.

Characteristic*	6 months^‡^		12 months^‡^		Mean change at 6 months from baseline (95% CI)			Mean change at 12 months from baseline (95% CI)		
	Pallidal stimulation	Subthalamic stimulation	Pallidal stimulation	Subthalamic stimulation	Pallidal stimulation	Subthalamic stimulation	*P* value	Pallidal stimulation	Subthalamic stimulation	*P* value
BFMDRS-M (range, 0–40)^§^	4.52 ± 2.83	3.88 ± 2.69	4.33 ± 2.78	3.50 ± 2.60	− 7.05 (− 8.94 to − 5.15)	− 5.76 (− 7.20 to − 4.33)	0.282	− 7.24 (− 9.20 to − 5.27)	− 6.14 (− 7.59 to − 4.70)	0.363
Eye(range, 0–8)	2.02 ± 1.42	2.36 ± 1.38	1.83 ± 1.30	2.07 ± 1.44	− 3.21 (− 4.19 to − 2.24)	− 3.43 (− 4.30 to − 2.56)	0.697	− 3.41 (− 4.51 to − 2.31)	− 3.71 (− 4.56 to − 2.87)	0.596
Mouth (range, 0–8)	1.74 ± 1.34	1.29 ± 1.55	1.62 ± 1.21	1.19 ± 1.49	− 2.21 (− 3.01 to − 1.42)	− 1.62 (− 2.43 to − 0.81)	0.292	− 2.33 (− 3.30 to − 1.36)	− 1.71 (− 2.52 to − 0.90)	0.328
Speech & swallowing (range, 0–16)	0.48 ± 0.93	0.05 ± 0.22	0.52 ± 0.98	0.05 ± 0.22	− 0.90 (− 1.68 to − 0.13)	− 0.52 (− 1.36 to 0.31)	0.446	− 0.86 (− 1.42 to − 0.29)	− 0.52 (− 1.36 to 0.31)	0.432
Neck (range, 0–8)	0.29 ± 0.77	0.19 ± 0.60	0.36 ± 0.94	0.19 ± 0.60	− 0.33 (− 0.83 to 0.17)	− 0.19 (− 0.59 to 0.21)	0.657	− 0.26 (− 0.64 to 0.12)	− 0.19 (− 0.59 to 0.21)	0.796
BFMDRS-D (range, 0–30)^§^	1.15 ± 1.18	0.55 ± 1.28	0.67 ± 0.91	0.35 ± 0.75	− 3.29 (− 4.67 to − 1.90)	− 3.62 (− 4.61 to − 2.63)	0.702	− 3.71 (− 5.21 to − 2.22)	− 3.81 (− 4.98 to − 2.64)	0.922
Speech (range, 0–4)	0.33 ± 0.58	0.00 ± 0.00	0.24 ± 0.44	0.00 ± 0.00	− 0.29 (− 0.48 to 0.01)	− 0.24 (− 0.50 to − 0.08)	0.771	− 0.33 (− 0.60 to − 0.07)	− 0.29 (− 0.50 to − 0.08)	0.771
Writing (range, 0–4)	0.14 ± 0.36	0.10 ± 0.30	0.00 ± 0.00	0.10 ± 0.30	− 0.81 (− 1.21 to − 0.41)	− 1.10 (− 1.55 to − 0.64)	0.343	− 0.95 (− 1.40 to − 0.51)	− 1.10 (− 1.57 to − 0.62)	0.658
Feeding (range, 0–4)	0.14 ± 0.36	0.10 ± 0.30	0.14 ± 0.36	0.05 ± 0.22	− 0.48 (− 0.71 to − 0.24)	− 0.71 (− 0.97 to − 0.46)	0.096	− 0.48 (− 0.71 to − 0.24)	− 0.76 (− 1.01 to − 0.52)	0.083
Eating and swallowing (range, 0–4)	0.19 ± 0.51	0.00 ± 0.00	0.19 ± 0.51	0.00 ± 0.00	− 0.24 (− 0.48 to 0.01)	− 0.05 (− 0.15 to 0.05)	0.104	− 0.24 (− 0.48 to 0.01)	− 0.05 (− 0.15 to 0.05)	0.104
Hygiene (range, 0–4)	0.00 ± 0.00	0.05 ± 0.22	0.00 ± 0.00	0.00 ± 0.00	− 0.24 (− 0.44 to − 0.04)	− 0.24 (− 0.44 to − 0.04)	1.000	− 0.24 (− 0.44 to − 0.04)	− 0.29 (− 0.50 to − 0.08)	0.748
Dressing (range, 0–6)	0.00 ± 0.00	0.00 ± 0.00	0.00 ± 0.00	0.00 ± 0.00	− 0.14 (− 0.31 to 0.02)	− 0.05 (− 0.15 to 0.05)	0.329	− 0.14 (− 0.31 to 0.02)	− 0.05 (− 0.15 to 0.05)	0.329
Walking (range, 0–4)	0.38 ± 0.50	0.29 ± 0.64	0.19 ± 0.40	0.19 ± 0.40	− − 1.05 (− 1.58 to − 0.52)	− 1.19 (− 1.53 to − 0.85)	0.642	− 1.24 (− 1.81 to − 0.66)	− 1.29 (− 1.72 to − 0.85)	0.895

*Plus–minus values are means ± SD.

^‡^Assessments with DBS on.

^§^A higher score indicates worse functioning.

**Figure 2:**
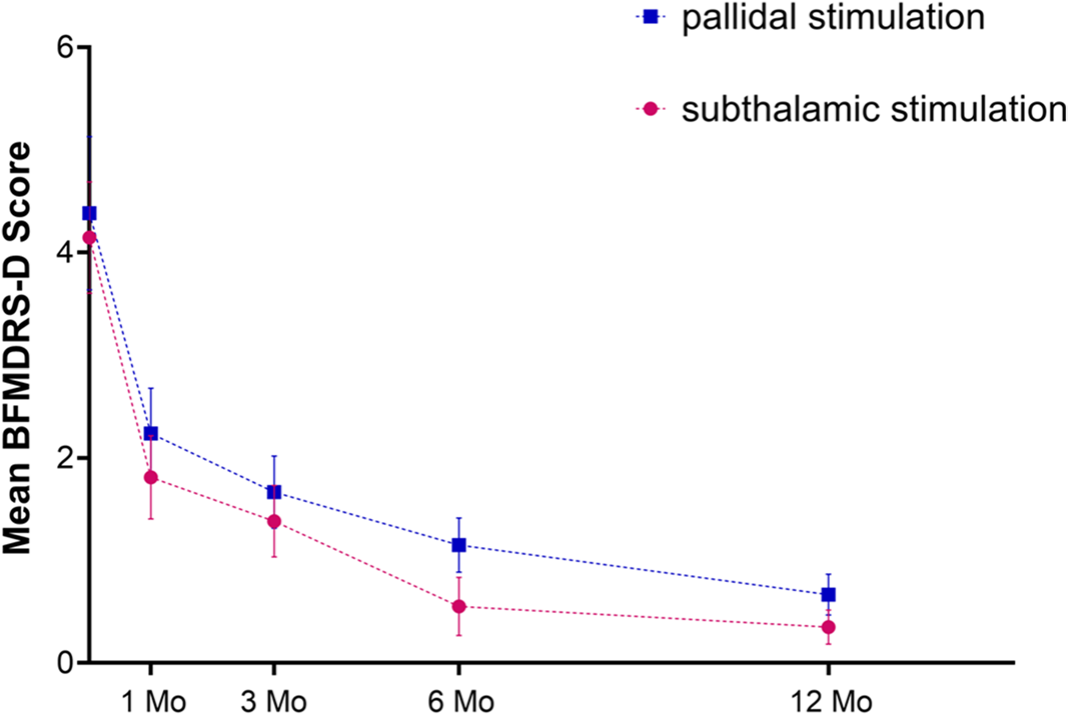
Burke–Fahn–Marsden Dystonia Rating Scale disability subscale scores (BFMDRS-D; scores range from 0 to 30, with higher scores indicating greater impairment) at baseline and throughout the 12-month study period. There was no between-group differences in the mean changes in BFMDRS-D scores based on the stimulation target. I bars indicate the standard error of the mean.

At 1 month after surgery, the mean score on the BFMDRS-D (highest possible score, 30) improved by 46.5% in the GPi group (from 4.19 ± 3.56 to 2.24 ± 2.02) and by 56.3% in the STN group (from 4.14 ± 2.50 to 1.81 ± 1.86). At 3 months, the score improved by 60.1% in the GPi group (from 4.19 ± 3.56 to 1.67 ± 1.62) and by 66.7% in the STN group (from 4.14 ± 2.50 to 1.38 ± 1.60) (Fig. 2, Table 2). At 6 months, the score improved by 72.6% in the GPi group (from 4.19 ± 3.56 to 1.15 ± 1.18) and by 86.7% in the STN group (from 4.14 ± 2.50 to 0.55 ± 1.28). At 12 months, the score improved by 84.0% in the GPi group (from 4.19 ± 3.56 to 0.67 ± 0.91) and by 91.5% in the STN group (from 4.14 ± 2.50 to 0.35 ± 0.75). We recorded no between-group differences in the mean changes in BFMDRS-D scores based on the stimulation target (Fig. 2, Table 3).

We examined how time and group influenced BFMDRS-M and BFMDRS-D results. Time was the only variable that showed an effect BFMDRS-M (*p* < 0.001), Eye (*p* = 0.007), Mouth (*p* = 0.014) and BFMDRS-M (*p* = 0.041). This indicates that there is a statistical difference in the change of scores in different time points. However, no statistical difference between groups (STN or GPI) was found in the ANOVA results (*P* > 0.05). Moreover, the results of time*group were also not statistically significant (*P* > 0.05), indicating that there is no interaction between time and group. (Table 4).

**Table 4:** Two factors ANOVA results in Primary Outcome (factors; time and group).

Characteristics	BFMDRS-M	Eye	Mouth	Speech & swallowing	Neck	BFMDRS-D	Speech	Writing	Feeding	Eating and swallowing	Hygiene	Dressing	Walking
*P* value *	< 0.001	0.007	0.014	0.257	0.997	0.041	0.400	0.136	0.101	0.410	0.993	1.000	0.116
*P* value^‡^	0.365	0.247	0.152	0.752	0.387	0.685	1.000	0.094	0.071	0.230	0.861	0.390	0.818
*P* value^§^	0.589	0.977	0.714	0.656	0.997	0.995	0.932	0.982	0.654	0.761	0.993	1.000	0.976

*Statistically analyzed using two factors ANOVA (factors; time).

^‡^statistically analyzed using two factors ANOVA (factors; group).

^§^statistically analyzed using two factors ANOVA (factors; time*group).

### Quality of life

**Figure 3:**
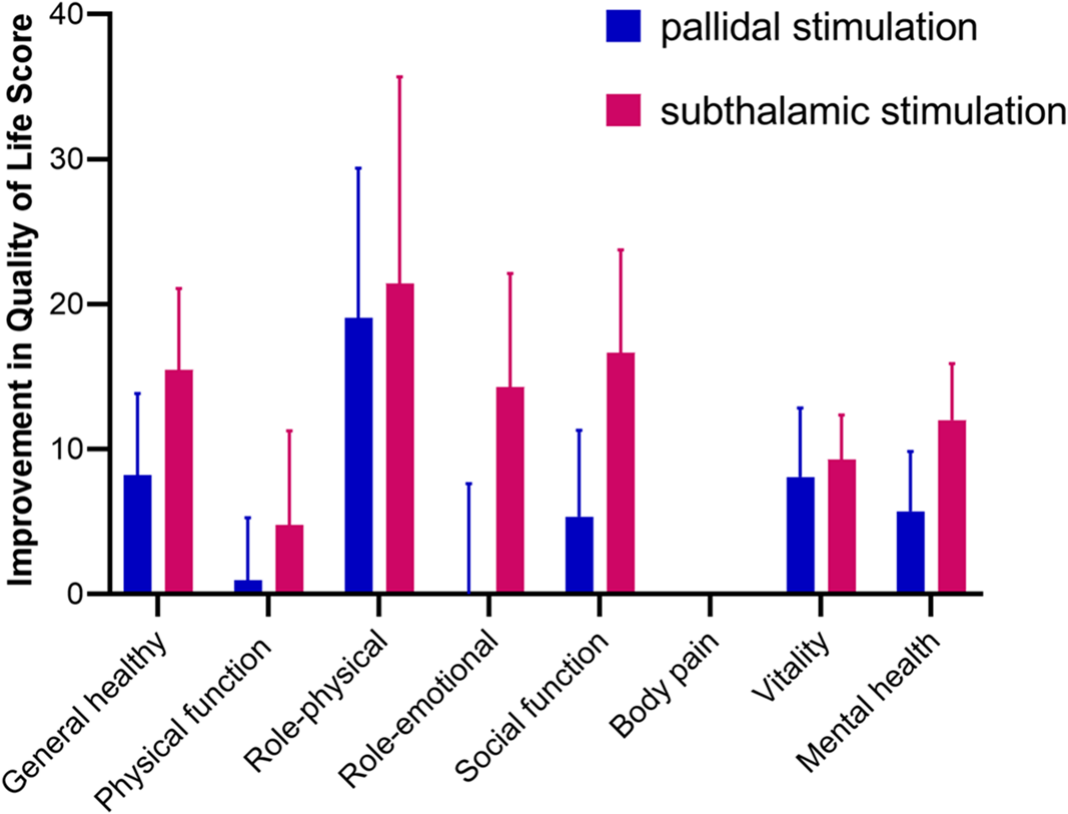
Scores for patient-reported quality of life on the Medical Outcomes Study 36-Item Short-Form General Health Survey (SF-36), with higher scores indicating better daily function and condition.

After 12 months after surgery, the quality of life improved on seven of eight subscales of the SF-36 in the two groups, with the exception of the level of pain that had not changed in either group. None of the between-group differences were significant (Fig. 3, Table 5). The mean changes in sleep PSQI scores (highest possible score, 21) at 12 months were 6.7% in the GPi group (from 13.48 ± 4.85 to 12.57 ± 4.25) and 9.7% in the STN group (from 13.05 ± 4.49 to 12.29 ± 4.60). We also found no statistically significant differences between the two groups (Table 5).

**Table 5:** Changes in Secondary Outcomes at 12 Months.

Outcome*	Pallidal stimulation		Subthalamic stimulation		Mean change at 12 months from baseline (95% CI)		
	Baseline	12 Mo^‡^	Baseline	12Mo^‡^	Pallidal stimulation	Subthalamic stimulation	*P* value ^†^
SF-36 scores¶							
General health (range, 0–100)	25.48 ± 12.14	33.69 ± 24.82	27.14 ± 10.67	42.62 ± 25.13	8.21 (− 3.51 to 19.94)	15.48 (3.78 to 27.17)	0.330
Physical function (range, 0–100)	77.38 ± 21.25	78.33 ± 23.79	72.62 ± 22.00	77.38 ± 24.12	0.95 (− 8.08 to 9.99)	4.76 (− 8.81 to 18.33)	0.614
Role—physical (range, 0–100)	38.10 ± 42.29	57.14 ± 47.53	40.48 ± 48.40	61.90 ± 46.52	19.05 (− 2.52 to 40.61)	21.43 (− 8.31 to 51.17)	0.869
Role—emotional (range, 0–100)	63.49 ± 40.70	63.49 ± 42.04	61.90 ± 39.84	76.19 ± 38.21	0.01 (− 15.91 to 15.92)	14.29 (− 2.04 to 30.61)	0.215
Social function (range, 0–100)	51.79 ± 20.27	57.14 ± 25.18	45.83 ± 14.97	62.50 ± 25.62	5.36 (− 7.05 to 17.77)	16.67 (1.90 to 31.43)	0.232
Body pain (range, 0–100)	100.00 ± 0.00	100.00 ± 0.00	97.86 ± 9.82	97.86 ± 9.82	0.00 (0.00 to0.00)	0.00 (0.00 to 0.00)	− −
Vitality (range, 0–100)	39.05 ± 14.80	47.14 ± 19.91	38.10 ± 7.98	47.38 ± 12.41	8.10 (− 1.79 to 17.98)	9.29 (2.88 to 15.69)	0.838
Mental health (range, 0–100)	41.52 ± 17.69	47.24 ± 21.71	46.29 ± 10.40	58.29 ± 16.76	5.71 (− 2.91 to 14.34)	12.00 (3.84 to 20.16)	0.331
17-item Hamilton Depression Rating Scale (range, 0–52)^§^	12.05 ± 6.25	11.71 ± 6.17	9.29 ± 4.01	5.90 ± 3.80	− 0.33 (− 1.72 to 1.05)	− 3.38 (− 5.44 to − 1.32)	0.014
14-item Hamilton anxiety rating scale (range, 0–56)^§^	14.76 ± 6.69	14.57 ± 6.43	11.14 ± 5.20	7.71 ± 5.07	− 0.19 (− 1.47 to 1.09)	− 3.43 (− 5.05 to − 1.80)	0.001
Pittsburgh Sleep Quality Index (range, 0–21)^§^	13.48 ± 4.85	12.57 ± 4.25	13.05 ± 4.49	12.29 ± 4.60	− 0.90 (− 1.81 to 0.00)	− 0.76 (− 1.73 to 0.20)	0.827

*Plus–minus values are means ± SD.

^†^* P* value s are for changes in scores from baseline to 12 months in the group undergoing pallidal stimulation, as compared with those undergoing subthalamic stimulation.

^‡^Assessments with DBS on.

^§^A higher score indicates worse functioning.

^¶^A higher score indicates better functioning.

### Depressive and anxiety

**Figure 4:**
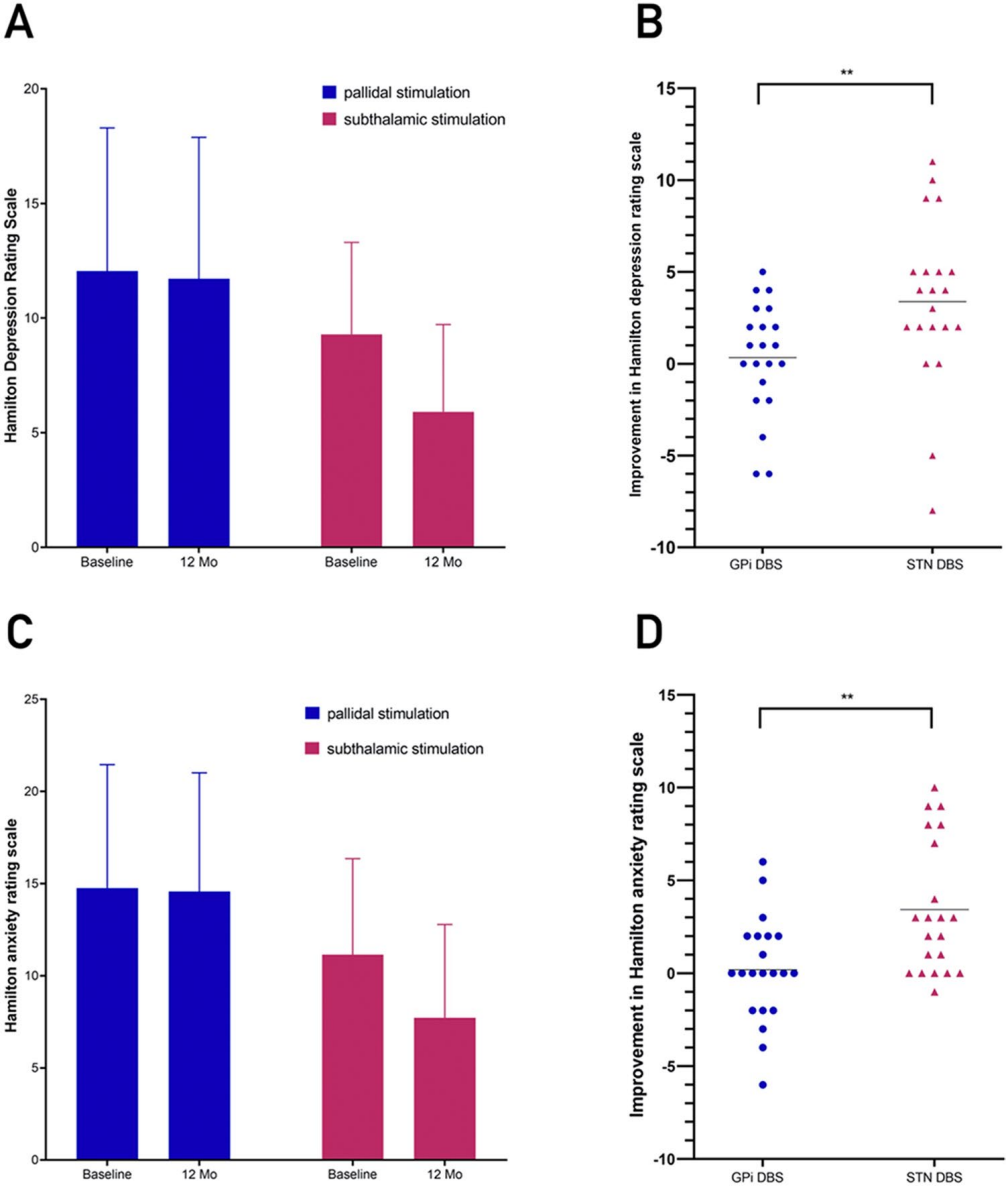
The effects of STN-DBS and GPi-DBS on depression and anxiety symptoms based on the 17-item Hamilton depression rating scale (HAMD) scores (scores range from 0 to 52, with higher scores indicating more severe depression) and 14-item Hamilton anxiety rating scale (HAMA) scores (scores range from 0 to 56, with higher scores indicating more severe anxiety). (A) HAMD scores for patients with STN-DBS and GPi-DBS at baseline and the last follow-up. (B) The comparison in efficacy for clinical improvement based on HAMD scores between STN-DBS and GPi-DBS. (C) HAMA scores for patients with STN-DBS and GPi-DBS at baseline and the last follow-up. (D) The comparison in efficacy for the clinical improvement based on HAMA scores between STN-DBS and GPi-DBS. "**" indicates *P* < 0.05.

On the 17-item HAMD (highest possible score, 52), the mean change from baseline significantly differed between the two study groups, with patients undergoing pallidal stimulation showing a 2.8% improvement (from 12.05 ± 6.25 to 11.71 ± 6.17, − 0.33 points) and patients undergoing subthalamic stimulation showing a 36.5% improvement (from 9.29 ± 4.01 to 5.90 ± 3.80, − 3.38 points) (*P* = 0.014) (Fig. 4, Table 5).

On the 14-item HAMA (highest possible score, 56), the mean change from baseline significantly differed between the two study groups, with patients undergoing pallidal stimulation showing a 24.5% improvement (from 14.76 ± 6.69 to 14.57 ± 6.43, − 0.19 points) and patients undergoing subthalamic stimulation showing a 47.1% improvement (from 11.14 ± 5.20 ± 6.43 to 7.71 ± 5.07, − 3.43 points) (*P* < 0.001) (Fig. 4, Table 5).

### Adverse events

A total of 14 adverse events occurred in 21 patients undergoing GPI-DBS and 7 adverse events in 21 patients undergoing STN-DBS (Table 6). There were no significant between-group differences in the frequency or type of serious adverse events. Except for stroke, all of these events were resolved by the conclusion of the 12-month study follow-up. In 14 (66.7%) patients undergoing pallidal stimulation and 16 (76.2%) patients undergoing subthalamic stimulation, no adverse events occurred during follow-up. Adverse events included vertigo in 1 patient (4.8%) in each of the two groups; dyskinesia in 1 patient (4.8%) in the GPi group and in 4 patients (19.0%) in the STN group; and paresthesias or numbness in 3 patients (14.3%) in the GPi group and in 1 patient (4.8%) in the STN group. The sensory side effects of numbness or paresthesia involved the face, hands, back and legs.

**Table 6:** Adverse Events at 12 Months.

Adverse Event*	Pallidal stimulation no. (%)	Subthalamic stimulation no. (%)	*P* value†
Vertigo	1 (4.8%)	1 (4.8%)	1.000
Dyskinesia	1 (4.8%)	4 (19.0%)	0.153
Paresthesia or numbness	3 (14.3%)	1 (4.8%)	0.293
Face	0	1 (4.8%)	
Hand and fingers	1 (4.8%)	0	
Back	1 (4.8%)	0	
Leg	1 (4.8%)	0	
Fall	1 (4.8%)	0	0.311
Stroke	0	1 (4.8%)	0.311
Disequilibrium sensation	1 (4.8%)	0	0.311
Dysphagia	2 (9.5%)	0	0.147
Fatigue	1 (4.8%)	0	0.311
Dysarthria	3 (14.3%)	0	0.072
Dyspnea	1 (4.8%)	0	0.311
No adverse events	14 (66.7%)	16 (76.2%)	0.495

*All events are listed according to the definitions used in the Medical Dictionary for Regulatory Activities, version 14.0, for serious adverse events and moderate or severe adverse events.

^†^χ^2^ or Fisher’ s exact test when appropriate.

In addition, the patients undergoing pallidal stimulation experienced disequilibrium sensation (1 patient; 4.8%), dysphagia (2 patients; 9.5%), fatigue (1 patient; 4.8%), dysarthria (3 patients; 14.3%), and dyspnoea (1 patient; 4.8%), which were not found among the patients in the STN group. However, 1 patient (4.8%) had a cerebral infarction after undergoing thalamotomy that was categorized as a serious adverse event (Table 6).

### Stimulation settings

At 12 months, the average stimulation amplitudes significantly differed between the group undergoing pallidal stimulation (4.30 V) and the group undergoing subthalamic stimulation (2.66 V) (*P* < 0.001); the average pulse widths were 95.24 μs and 61.67 μs, respectively (*P* < 0.001); and frequencies were 163.33 Hz and 130.95 Hz, respectively (*P* < 0.001).

## Discussion

DBS improved motor function in patients with Meige syndrome who underwent either GPi or STN stimulation, with no significant differences in improvements between the two surgical targets during the 12 months of follow-up, as assessed by scores on the BFMDRS-M in patients who were not taking medication. There was also no significant differences in improvements on the four subscale scores on the BFMDRS-M between the two groups during the 12-month follow-up. Furthermore, our results showed no differences in improvement between the GPi and STN targets in terms of BFMDRS-D scores, SF-36 scores and PSQI sleep scores. In secondary analyses, however, STN-DBS was associated with better improvements in depression and anxiety symptoms than GPi-DBS. Both DBS targets did not cause greater cognitive, mood, or behavioural side effects. Our previous studies^5,6^ have shown that GPi-DBS or STN-DBS are effective and safe treatments for Meige syndrome. Although some results about DBS-GPi have already been reported in our recent study in 2020^5^, the new and different matters in the present study is a comparison between the two methods. Although there have been some studies on the advantages and drawbacks of GPi and STN as neurostimulation targets in the treatment of Meige syndrome^7,8^. These studies were limited because an even larger clinical sample is required to establish clear conclusions and did not examine psychological elements. Our study is the first to compare the efficacy of stimulating these targets on the motor and non-motor symptoms of Meige syndrome with large clinical sample. This study is a retrospective study, but all data used in this study have been carefully recorded by documents or videotapes. For the data of GPi-DBS, we need to clarify that the included patients in this study are not exactly the same as our paper published in 2020^5^, due to the reason of missing follow-up and informed consent of some patients. In addition, experts who were not involved in the treatments were recruited to objectively evaluate the clinical outcomes from videotape analysis. Therefore, our study has high accountability in assessing and comparing the efficacy of DBS treatment for Meige syndrome.

Meige syndrome, also known as blepharospasm-oromandibular dystonia syndrome, was first described and named after French neurologist Henri Meige in 1910^9^. It is a rare segmental cranial-neck dystonia. This disease starts insidiously, develops slowly, involves mainly the eyelid, mouth and jaw and neck muscles, and seriously affects the patient's work and life^10^. Patients with early, mild disease can choose drug therapy first. Single drug therapy, such as anticholinergic or benzodiazepine therapy, often has limited efficacy and requires the combined use of multiple drugs^11^, and its use is limited by serious side effects^12^. Although local injection of botulinum toxin can effectively relieve muscle spasm, with nerve regeneration and toxin metabolism, transmitter delivery and muscle function can gradually be restored, leading to the recurrence of dystonia symptoms^13^. DBS is a new treatment method for Meige syndrome based on the development of stereotactic technology^4,6,8,14–19^. Guided by MRI, microelectrodes are implanted into the GPi or STN to continuously stimulate the brain to relieve the symptoms of dystonia^20^. In our previous studies^5,6^, it had been demonstrated that GPi-DBS or STN-DBS plays an important role in the treatment of Meige syndrome, and its curative effect is definite and lasting. Moreover, with the continuation of stimulation, the improvement in patients' motor symptoms and functional dysfunction becomes more obvious. This is also consistent with the results of this study, in which bilateral GPi-DBS and STN-DBS reduced the BFMDRS-M scores by 61.3% and 63.7% and BFMDRS-D scores by 84.0% and 91.5%, respectively, at 12 months of neurostimulation.

Previous studies^21,22^ on the treatment of other diseases by DBS have shown that there are differences between the two DBS targets in terms of the effects, difficulty of the operation and parameter setting for improving motor and non-motor symptoms. In a study by Wang et al.^23^ based on the literature of 75 patients with Meige syndrome treated with DBS, there was no significant difference in efficacy between GPi-DBS and STN-DBS (67.3% vs. 51.6%). However, this study was a retrospective study based on the literature, and Only 6 STN patients included. In addition, the minimum follow-up time was only 2 months. In this study, we did not observe a significant difference in improvements in motor function during 12 months of follow-up in the two treatment groups. At the same time, there was also no statistically significant differences in the improvements in blepharospasm, oromandibular dystonia or cervical dystonia symptoms (four subscales of the BFMDRS-M scores) in Meige syndrome between the two groups during the 12-month follow-up. However, after 1 month, the BFMDRS-M improvements in scores were nearly twice as much in the STN group as in the GPi group (41.2% vs. 26.4%), although there was no statistically significant difference. Our results indicated that although both STN and GPi stimulation can improve Meige syndrome within 1 month after surgery, the rapid motor improvement gained may be greater with STN-DBS than with GPi-DBS. This phenomenon is in line with an isolated dystonia study by Lin et al., who described that the percentage improvement in the BFMDRS-M score was significantly larger after STN-DBS (64%) than after GPi-DBS (48%) at the 1-month follow-up.

Non-motor function is an important determinant of quality of life in patients with Meige syndrome and should be considered during DBS target selection^5,6^. In this study, significant differences between the two study groups on measures of non-motor function were limited to depressive and anxiety symptoms on the 17-item HAMD and 14-item HAMA, and we did not observe a significant difference between the two study groups in quality of life, as measured by the SF-36 and in sleep quality status, as measured by the PSQI. In a primary dystonia study, comparable motor improvements with similar quality of life improvements at 1 month were observed; all eight domains on the SF-36 showed significant improvements in both groups^22^. This is consistent with the results of this study, with the exception of improvements in the domain of pain. Only one patient in the STN group of all 42 patients had preoperative symptoms of ocular pain, and pain symptoms did not improve at the end of follow-up despite improvement in ocular dystonia. Since patients with Meige syndrome rarely have disease-related pain^9^, including more patients with pain or extending the follow-up period will be important to determine responses to stimulation at the two targets. In addition, in our previous two studies^5,6^, patient sleep quality was significantly improved after STN-DBS treatment but did not experience amelioration after continuous GPi-DBS in another study. In this study, we did not observe a significant difference between the two study groups in sleep quality status, as measured by the PSQI. These results are consistent with studies comparing improvements in sleep quality in patients with Parkinson's disease receiving stimulation of the STN and GPi^24,25^. STN-DBS and GPi-DBS may improve patients' sleep by affecting sleep–wakefulness regulatory centres^26^ and cortico-striato-pallido-thalamic circuits^27^, respectively. The STN is located at the diencephalo-mesencephalic junction, posterolateral to the hypothalamus, and medial to the substantia nigra and red nucleus^28^. It is thus not far away from wake-promoting midbrain areas^29^. The nucleus has inhibitory connections to the anterior hypothalamus and the upper part of the mesencephalic reticular substance^30^, and glutamatergic innervations to the substantia nigra pars compacta which in turn innervate several brain areas involved in sleep regulation^31^. In addition, part of the basal ganglia interconnecting the cortico-striato-pallido-thalamic circuits might be involved in sleep regulation^27^. Finally, depression and anxiety are widely found in patients with dystonia^32^ and have also been reported in patients with Meige syndrome^33^. Our previous study^6^ showed that GPi-DBS did not relieve depressive symptoms in patients with Meige syndrome. The present study showed greater improvements in depression and anxiety symptoms based on HAMD and HAMA scores in the STN-DBS group than the GPi-DBS group (36.5% vs. 2.8% and 47.1% vs. 24.5%, respectively), which extends our previous finding. It is possible that STN-DBS stimulation could influence brain structures responsible for mood. STN has limbic territories, and spread of stimulation to these sites could influence depression^34^.

The average stimulus amplitude, pulse width and frequencies in the STN-DBS group patients were lower than those in the GPi-DBS group patients, and the time interval for replacing the pulse generator might be longer for STN-DBS patients, which made the long-term treatment cost lower and reduced the surgical risk associated with replacing the pulse generator^35,36^. The difference in amplitudes we observed was consistent with a randomized study involving Parkinson patients undergoing either pallidal stimulation or subthalamic stimulation, in which average amplitudes for the two types of neurostimulation differed by 0.8 V at 1 year after the operation^37^. Voltage stimulation was lower in STN-DBS than in GPi-DBS, likely due to the smaller dimension of the STN and the proximity to other relevant structures in the midbrain^38^. In contrast, higher electrical stimulation settings can be observed in GPi-DBS for PD, presumably due the large volume of the pallidum^39^. However, we believe that advances in pulse generator technology may make this factor less important in the future.

Taking all these factors into consideration, the primary outcomes measured and based on BFMDRS-M and BFMDRS-D scores were not significantly different based on DBS targets, so we could not conclude that one target was superior to another based on these measures. Our study showed that both DBS targets are feasible in terms of improving motor symptoms in patients with Meige syndrome. However, in addition to focusing only on improvements in motor function, clinicians also need to include quality of life and non-motor symptoms, such as sleep, depression and anxiety, during the selection of DBS targets. Our data suggest that STN-DBS was associated with better improvements in depression and anxiety symptoms than GPi-DBS.

## Limitations

Some limitations must be considered in our study. First, our study was a retrospective study. A randomized controlled trial should be performed in the future. However, as mentioned earlier, this study was the first controlled clinical study of STN-DBS and GPi-DBS for Meige syndrome. All data were carefully preserved in video or documentation and evaluated by a senior neurologist who was not involved in the entire treatment process. Therefore, the results of this study are very reliable and of great significance for the selection of DBS targets in the treatment of Meige syndrome. Second, we investigated the effects of DBS targets on sleep quality, a sleep efficiency-related study in Meige syndrome patients with insomnia remains to be conducted. The effect of the two DBS targets on other non-motor symptoms, such as psychiatric and cognitive symptoms, should be assessed in future studies. Third, the mean follow-up time in this study was 1 year, so extended follow-up will be important to determine responses to stimulation at the two targets.

## Conclusions

Patients with Meige syndrome had similar improvements in motor function, quality of life and sleep after either pallidal or subthalamic stimulation. Depression and anxiety factors may reasonably be included during the selection of DBS targets for Meige syndrome. STN-DBS was associated with a better improvement in depression and anxiety symptoms than GPi-DBS.

## Methods

### Patients

Forty-two Asian patients with primary Meige syndrome who underwent neurostimulation of the GPi or STN were collected between September 2017 and September 2019 at the Department of Neurosurgery, Peking University People’s Hospital. The diagnostic criteria were mainly based on blepharospasm, oromandibular dystonia and cervical dystonia, increased blink rates and other symptoms^40^.

The inclusion criteria were as follows: 1. Primary Meige syndrome was diagnosed by an experienced neurologist, Ruen Liu. 2. All patients had received systematic and regular treatment for at least 1 year before surgery, including oral drugs and local injection of botulinum toxin A, but the efficacy was not apparent, as the ability to engage with daily life and the quality of life of the patients significantly decreased. 3. There were no other serious systemic diseases, such as severe organic heart disease, severe lung, liver and kidney dysfunction, and coagulation dysfunction. 4. There was no history of neurological diseases other than Meige syndrome, such as Parkinson's disease or severe cognitive dysfunction. 5. There were no serious psychiatric disorders, such as schizophrenia. 6. Preoperative head magnetic resonance imaging (MRI) examinations were normal. Patients of missing follow-up and incomplete clinical data were excluded from the cases.

The authors ensure the accuracy and completeness of the data and data analysis. The first author wrote the first draft, and all authors decided to submit the manuscript for publication. Written informed consent was obtained from each participant, and the study was approved by the institutional review board of Peking University People’s Hospital. The ethical approval number was “2020PHB065-01”. All experiments were performed in accordance with relevant guidelines and regulations.

### Surgical procedures and DBS programming

The surgical procedure for bilateral STN-DBS or GPI-DBS was consistent with the previous study from our team^5^. In brief, the patient underwent bilateral stereotactic surgery under local anaesthesia. The globus pallidus interna or subthalamic nucleus was located by combining stereotactic MRI with microelectrode recording. The GPi is located the front 2–3 mm from the midpoint of the anteroposterior commissure, 18–22 mm lateral, and 6–9 mm below the plane of the anteroposterior commissure. The STN is located 2–3 mm behind the midpoint of the anteroposterior commissure, 12–14 mm lateral, and 4–6 mm below the plane of the anteroposterior commissure. The DBS electrode (model L302, PINS Medical, Beijing, China) and the pulse generator (G102R, PINS Medical) were implanted, and the final position of the electrode was confirmed by neuroimaging. One month after surgery, the stimulation was initiated. The optimal stimulation settings were progressively adjusted according to the patient’s response. The standard pulse setting was 60 μsec in duration at 130 Hz, with the voltage adjusted to the individual patient. In addition, based on each patient’s response to neurostimulation, the parameters could be progressively adjusted at outpatient follow-up or by a telemedical application.

### Evaluation and follow-up

The primary outcome was the change in motor function. Burke–Fahn–Marsden Dystonia Rating Scale scores were obtained for the movement (BFMDRS-M) and disability (BFMDRS-D) subscales^41^ (scores can range from 0–40 to 0–30, respectively, with higher scores indicating greater impairment), based on evaluation of video recordings (Videos 1 and 2) obtained at specific time points: 3 days before DBS (baseline) surgery and 1, 3, 6, and 12 months after surgery. The severity of dystonia in each patient was evaluated by an independent movement disorder neurologist (Hu Ding) who was not involved in the surgery or DBS programming. Secondary outcomes included health-related quality of life, sleep quality status, depression severity, and anxiety severity obtained at 3 days before DBS (baseline) surgery and 12 months after surgery. Patient health-related quality of life was assessed with a validated version of the Medical Outcomes Study 36-Item Short-Form General Health Survey (SF-36)^42^, which covers 8 aspects of the health status of each patient; scores on each scale can range from 0 (worst) to 100 (best), with higher scores indicating better daily function and condition. The sleep quality status of the patients was assessed using the Pittsburgh Sleep Quality Index (PSQI). The PSQI total score can range from 0 to 21. The higher the score, the worse the quality of sleep^43^. A trained rater assessed depressive symptoms using the 17-item Hamilton depression rating scale (HAMD)^44^ (scores can range from 0 to 52, with higher scores indicating more severe depression) and assessed anxiety symptoms using the 14-item Hamilton anxiety rating scale (HAMA) (scores can range from 0 to 56, with higher scores indicating more severe anxiety).

### Adverse events

The patients were queried about adverse events by the neurologist (Dongliang Wang), and adverse events in all patients were reported and coded according to the Medical Dictionary for Regulatory Activities, version 14.1 (categorized as mild, moderate, or severe in intensity). A serious adverse event was defined as any event that resulted in death, disability or prolonged or new hospitalization and causes serious damage to health.

### Statistical analysis

Scores on the movement and disability subscales of the BFMDRS before and after the intervention were our primary outcome measures. The secondary outcome measures were health-related quality of life (SF-36 scores), sleep quality status (PSQI scores), depression severity (HAMD scores), anxiety severity (HAMA scores) and adverse events.

The time of evaluation was treated as a categorical variable. Differences between study groups were compared by hypothesis tests. Statistical significance between the quantitative variables was assessed by χ^2^ tests, with Yates's or Fisher's correction, if necessary. Student's t-tests were performed to evaluate data that followed a normal distribution. Bonferroni correction was applied for multiple comparisons. For primary outcomes (BFMDRS-M and BFMDRS-D), comparison between two groups at each time points were statistically analyzed using two factors ANOVA (factors; time and group). Significant differences between groups were indicated when *P* < 0.05. Numerical variables are expressed as the mean ± SD. Qualitative variables are described as the absolute value of cases in the distinct group. Statistical analyses were performed using SPSS 25.0 (IBM Corp., Armonk, NY, USA).

## Supplementary Information

Supplementary Information 1.

Supplementary Video 1.

Supplementary Video 2.

## Data Availability

Data collected for the study, including individual participant data and a data dictionary defining each field in the set, will be made available to others; deidentified participant data, participant data with identifiers, data dictionary, or other specified data set will be made available; study protocol, statistical analysis plan, informed consent form, and other related documents will be available; with publication, these data will be available; these data can be obtained by contacting the corresponding author Ruen Liu at the email address liuruen@pku.edu.cn; with investigator support, after approval of a proposal and with a signed data access agreement, data will be shared.
