# Supplementary material for: Pallidal versus subthalamic deep-brain stimulation for meige syndrome: a retrospective study
Source: Sci Rep. 2021 Apr 22;11:8742. doi: 10.1038/s41598-021-88384-4 (PMC8062505; doi:10.1038/s41598-021-88384-4)
Supplement: Supplementary file 1 — Supplementary Information 1. [file 41598_2021_88384_MOESM1_ESM.docx]

VIDEO 1. Before surgery: video clip showing a patient who has had Meige syndrome for 11 years. She had severe blepharospasm, with continuous involuntary movements of the jaw and mouth muscles and an involuntary left twist of the neck. The ability to engage in daily life and the quality of life of the patients had significantly decreased. The patient in Videos 1 and 2 agreed to a release of her video without masking. Copyright, Hu Ding. Published with permission.

VIDEO 2. One month after surgery with continual neurostimulation, the patient returned to a near normal status. The patient in Videos 1 and 2 agreed to a release of her video without masking. Copyright, Hu Ding. Published with permission.
